# Yawning and airway physiology: a scoping review and novel hypothesis

**DOI:** 10.1007/s11325-022-02565-7

**Published:** 2022-02-05

**Authors:** Christiaan Jacob Doelman, Johannes Adriaan Rijken

**Affiliations:** Department of Head and Neck Surgical Oncology, University Medical Center Utrecht, Utrecht, the Netherlands

**Keywords:** Yawning, Gaping, Airway physiology, Airway patency, Airway lumen, Pharynx, Muscle physiology, Evolution, Behavioral science, New hypothesis

## Abstract

**Background and purpose:**

Yawning is a stereotypical complex muscular movement and is commonly executed by most vertebrates. In seconds, the entire airway is fully dilated and surrounding muscles are powerfully stretched, most prominently around the pharynx. To date, yawning has been rarely studied, and as of yet there is no consensus on its main function.

**Material and methods:**

To investigate a mechanical airway function for yawning, a literature search was conducted to relate the frequency of yawning and obstructive airway conditions.

**Results:**

The results show that changes in obstructive airway conditions and alteration of the frequency of yawning are temporally related.

**Interpretation:**

These relationships, however, cannot be interpreted as causal, nor can they be extrapolated to explain the function of yawning. Yet airway management and yawning share many physiological characteristics. We therefore propose a novel hypotheses: yawning plays a significant role in airway physiology by muscle repositioning and widening the airway lumen, thereby securing long-term oxygenation.

## Introduction

### General characteristics

Yawning, gaping, or oscitation is a common and stereotyped physical behavior performed by most vertebrates, including mammals, birds, reptiles, amphibians, and even fish [1, 2]. In most animals, a regular yawn lasts 4 to 7 s, and is characterized by (1) a long inspiratory phase with gradual mouth gaping, followed by (2) a brief climax (or acme) with powerful muscle stretching, and (3) a rapid expiratory phase with muscle tension release [1, 3, 4]. For fish and birds, this is described as gradual mouth gaping, staying open for at least 3 s and subsequently a rapid closure of the mouth [5]. A yawn is performed from fetal stages to old age, in human fetuses starting from the 11th or 12th post-conceptional week [6, 7].

### Frequency of yawning

Fetuses yawn around 25 times per day, [8] after which the frequency decreases with age [9]. Adults have been observed to yawn about nine times per day (range 0–28) [10], often in fits of two or three with increasing intensity [3, 11]. Yawning frequencies of animals have hardly been studied, but convey the impression of being similar [12, 13]. Yawning’s frequency is influenced by multiple internal and external triggers. Primarily, a yawn is triggered in a low-vigilance state of the brain while transiting between wake and sleep. Both the awaking process and progressive drowsiness regularly provoke a yawn [1, 9, 14]. Particular stressful events (e.g., athletes before a game), imitation of a yawn, and hungriness have also been reported as endogenous triggers [3, 10]. In humans and certain social animals, yawning is sometimes triggered by exogenous triggers: seeing or hearing another individual yawn, or reading about yawning, which you may have just experienced [5, 15, 16]. This is defined as contagious yawning and can be suppressed with difficulty. Opioid withdrawal syndrome, psychoactive drugs (e.g., apomorphine, naloxone after morphine), and neurological diseases (e.g., amyotrophic lateral sclerosis, multiple sclerosis) are related to excessive yawning, which is defined as more than 3 yawns per 15 min [17, 18]. Opioid peptides are known to inhibit a yawn, [19] and some psychotic disorders may be related to decreased yawning, of which no cutoff value has so far been reported [20].

### Basic neurology of yawning

**Fig. 1:**
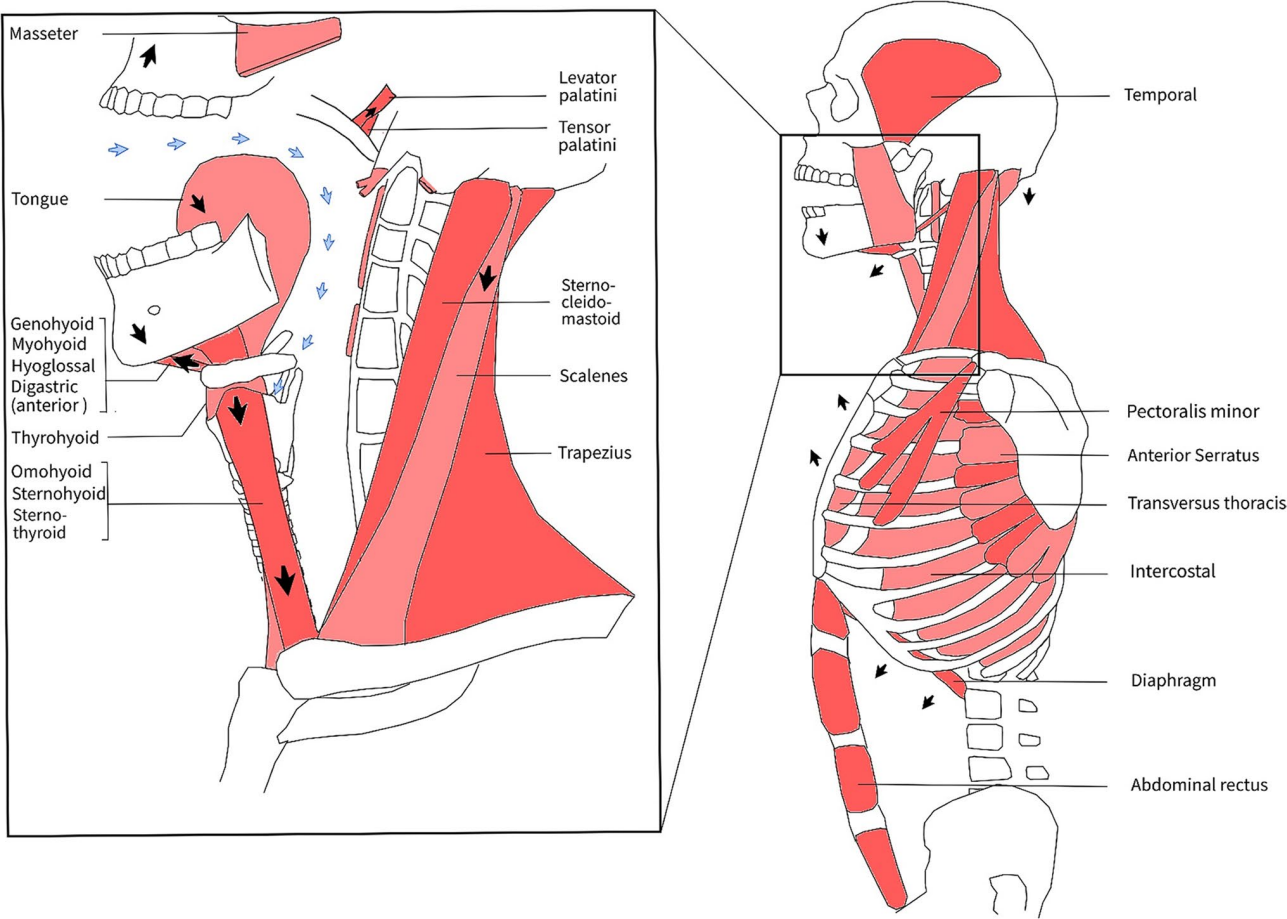
Muscle movements during a yawn: anatomical overview. A yawn can be divided in three phases: (1) the inspiratory phase in which the respiratory muscles gradually contract, (2) the climax (or acme) phase with maximal muscle stretching, and (3) the expiratory phase with muscle relaxation and a satisfied sensation. During the first phase, the subhyoidal muscles (thyrohyoid, sternohyoid, sternothyroid, omohyoid) and floor of mouth muscles (geniohyoid, mylohyoid, hyoglossus, and anterior bellies of the digastric) gradually contract, resulting in jaw opening and pharyngeal dilation (3–4 × diameter). Thereby, the diaphragm, intercostals, and additional respiratory muscles (e.g., scalene muscles, minor pectoral) contract causing deep inspiration (blue arrows). Peak forces are achieved during the second phase, where jaw (e.g., masseter and temporal), pharyngeal (Fig. 3), and other body muscles (e.g., arms, trunk) are powerfully stretched (Fig. 3). Afterwards, prolonged maximal dilation and inspiration muscle tension are released and expiration follows (third phase)

Yawning is a complex neurological act, mainly orchestrated in the brainstem near the basic life centers for breathing, swallowing, mastication, and coughing [19]. Top-down control by cortical, limbic, and hypothalamic centers is involved in triggering yawning and voluntary inhibition in certain intelligent animals [19]. A variety of neuroactive agents have been identified to contribute to a yawn: nitric oxide, dopamine, acetylcholine, glutamate, serotine, adrenocorticotropic hormone (ACTH), oxytocin, and steroid hormones [19]. Based on lesion studies and neuropharmacological studies, effector neurons are thought to be the cranial nerves V, VII, IX, X, XI, and XII (innervating the masseter, facial, pharyngeal, laryngeal, neck, and tongue muscles respectively), the cervical nerves (innervating the diaphragm and scalene muscles), and thoracic nerves (innervating the intercostal muscles) [19, 21]. These neurons fire in a unique rhythmical order, resulting in a complex pattern of contracting muscles around the respiratory and most proximal digestive tract (anatomical overview in Fig. 1).

*Observe your own yawns in the mirror. If not already triggered, one can voluntarily provoke a yawn by inhaling gradually while retracting the tip of the tongue and opening the jaw repetitively.*

### The act of yawning

At first, gradual inspiration is caused by contraction of the diaphragm and intercostal muscles. Then, the mandible lowers, and the mouth may open, defined as “gaping” (one can yawn without opening the mouth; however, this is less satisfactory). The tip of the tongue retracts and moves downward, accompanied by prominent downward movement of the larynx and hyoid bone, which is achieved by contracting infrahyoidal muscles (Fig. 3) [3, 22]. Subsequently, the inspiration accelerates and the soft palate and posto-lateral side of the pharynx elevate, which is the point where a turbulent inhaling sound of the palate and clicking sounds of the opening eustachian tubes may be experienced [23]. At this point, the pharynx diameter has increased 3–4 times [1, 3, 24] and the hyoid bone has reached its nearest position to the mandible. Peak forces of dilator muscles (agonists) initiate the acme phase, where jaw and pharyngeal muscles (antagonists) are maximally stretched, often accompanied by stretching of other body muscles (e.g., trunk, arms) [3, 25]. This is also known as the stretch-yawning syndrome, or pandiculation (in Latin *pandare* = stretching), which is primarily observed when awakening [3]. This powerful stretching and maximal dilation of the airway from the mouth to alveolus takes 1 s or less and comprises the acme phase [26]. A facial grimace, closed eyes, and sometimes lacrimation might be observed. After the climax, muscle tension is released and expiration follows. This may be experienced with vocalization and a rewarding sensation [27]. The morphology of a yawn may, however, depend on the (social) context [28].

### Hypotheses of yawning’s function

The function of yawning’s complex motor pattern is widely debated. Four popular theories describe the function of yawning and indicate there is no consensus about yawning’s main function. First, the “brain arousal hypothesis” suggests yawning diffusely activates the brain [1]. However, brain arousal by yawning is not evident in EEG, skin conductance, and other autonomic parameters [29]. If present in studies, arousal might well be triggered by voluntary body movements [30]. Second, the “respiration hypothesis” describes yawning as an oxygenating maneuver, primarily to supply the brain [31]. This theory has been discarded since Provine et al. (1987) could not trigger yawning in students by exercise and by breathing through a hand-held mask with increased CO_2_ levels (5%). Likewise, stimulated yawns could not be inhibited by high (100%) O_2_ levels. This study mentioned no O_2_ or CO_2_ pressures in participants, but significant changes were not expected by Krestel et al. (2018). Therefore, a possible oxygenation function would not explain (frequent) yawning in utero. This oxygenating theory underlies the theory thata yawning supports short-term oxygenation (or saturation), while we suggest yawning enables ventilation by widening the airway, which may be interpreted as long-term oxygenation (see “Discussion”). Third, the “communication hypothesis” is based on the contagiousness of yawning and advocates for a communicating or group synchronization function [27]. Social animals only yawn contagiously for around 10% of episodes [14], while many other animals do not yawn by external stimuli at all [19]. Social interaction as a main function of yawning is not therefore supportable, though social interaction may well co-exist as a minor function. Fourth, the “brain cooling hypothesis” suggests the brain temperature decreases with yawning [32]. A recent publication of Massen et al. (2021) supported this hypothesis by relating the frequency of yawning with brain mass [2]. However, a significant decrease in temperature during a yawn is physically impossible according to critical review and calculations by physiologist Elo [33].

It is true that yawning has been studied to a limited extent compared to other behaviors. Due to the complex underlying mechanics of yawning, the neurological and behavioral science, and biological influences, it is challenging to determine an unambiguous function. By conducting an anatomical and literature study, together with inductive (bottom-up) reasoning, we propose a new hypothesis on the main function of yawning (see “Discussion”).

## Methods

To investigate a relationship between yawning and airway physiology, a systematic literature search was conducted to link obstructive airway conditions (pathology) to the frequency of yawning. As there is a little literature available, a broad definition of obstructive airway conditions was applied: symptomatic higher airflow resistance due to swelling, foreign bodies, tight pharyngeal muscles, and airway collapse as a result of muscle tension loss (obstructive sleep apnea or anesthesia). The frequency of yawning was defined without cutoff value: an observed increase or decrease of yawning. A scoping review study design was designated due to limited data. Furthermore, other features of yawning (e.g., deepness of a yawn or different patterns of a yawn) were not documented. Therefore, only the frequency of yawning was within the scope of this literature search. Two possible relationships were within the scope of this literature study: that the frequency of yawning (determinant 1) influenced obstructive airway conditions (outcome 1), and vice versa: obstructive airway conditions (determinant 2) influenced yawning frequency (outcome 2).

In June 2021, Medline (PubMed) and Embase searches were performed with the search term “yawning” combined with obstructive airway conditions using a combination of MeSH headings and keywords (Table 2 in Appendix 1). “Oscitation” and “gaping” (mouth opening) were not added to the search, as both are rarely used to describe yawning and cannot be found in PubMed’s MeSH terms of “yawning.” Results were uploaded and duplicates were removed in RefWorks 3 (ProQuest, Ann Arbor, MI, USA) [34]. Titles and abstracts were read twice at different dates by author CD. The following inclusion criteria were applied: English, human or animal studies relating the frequency of yawning with symptomatic obstructive airway conditions from 1 January 1950 to 1 June 2021. Exclusion criteria included letters to editors, congress abstracts, hypothesis studies, and reviews. Studies with determinant and outcome 1 and in which airway obstruction or higher airflow resistance was not clearly described (not able to be differentiated from physiological airway conditions) were excluded. Studies with determinant and outcome 2 and which did not describe a clear change in the frequency of yawning (not able to differentiate from normal or occasional yawning) were excluded as well. Second, studies with determinant and outcome 2 were excluded which as they may have had major confounding factors influencing the yawn frequency (e.g., opioid administration, micro-injections in the brain, or patients with central neurological diseases).

Complete articles of all citations resulting from this search were obtained*.* Additionally, ASReviews, [35] which is an algorithm designed to find related studies by deep learning, was applied to find extra articles that were not found in the initial search. The included articles served as input, beside assessment of 10 random Embase “yawning” results, after which full texts were analyzed of the top 50 most related articles of all articles about “yawning” on Embase.

Level of evidence was assessed using the table of LEGEND of the Cincinnati Children’s Hospital [36]. Critical appraisal was not implemented in this scoping review study design [37]. Study characteristics and statements relating yawning and airway obstructive pathology of the selected articles were obtained and summarized in Table 1.

**Table 1:** Studies relating yawning frequency and obstructive airway pathologies

Study author (year)	Study design	Level of evidence	Study methods (summarized)	Study results (concerning yawning + airway)	Temporal relationships	Possible confounding factors***
Bartlett (1971) [38] *	RCT	2b	100 patients after elective surgery: Group A (*n* = 50) used a spirometer combined and provoked yawning (at least 10 per hour). Group B (*n* = 50) control group	Intervention decreases postoperative pulmonary complications (e.g., atelectasis) from 30% (group B) to 10% (group A). Pulmonary complications reduced significantly	↑ Yawns ⥬ ↓ Obstructive airway (↓ Yawns ⥬ ↑ Obstructive airway)	Spirometer
Evans (1978) [39]	Case report	5b	2 case reports of chocking children in emergency setting with increased yawning	Increased yawning while choking; decreased yawning after the obstruction was relieved Yawning might be stimulated by vagal feedback arising from the pharynx	↑ Obstructive airway ⥬ ↑ Yawns ↓ Obstructive airway ⥬ ↓ Yawns	
Mukai (1991) [40]	Case series	4a	51 patients (age 3–59 weeks) with ankyloglossia were examined: O_2_ measurements, fibroscopic examination of epiglottal, and laryngeal deviation. Frenotomy and correction of tongue, epiglottis, and larynx if necessary	More frequent yawning in epiglottal and laryngeal deviation and when O_2_ levels dropped	↑ Obstructive airway ⥬ ↑ Yawns	Ankyloglossia sleepiness
Kim (2002) [41]	Randomized observational clinical study	2a	60 healthy adult patients were induced in anesthesia with thiopental (*n* = 30) and propofol (*n* = 30) and all patients were observed (verbal response, eye-lash reflex yawning, apnea) before intubation	During induction, 83% of thiopental patients and 63% of propofol patients yawned before intubation	↑ Obstructive airway ⥬ ↑ Yawns	Propofol, thiopental, sleepiness
Kasuya (2005) [42]	Prospective cohort	2a	60 patients ASA 1 / 2 undergoing elective surgery induced thiopental and propofol, together with atropine and hydroxyzine hydrochloride	During induction 60% of thiopental patients and 47% propofol patients yawned	↑ Obstructive airway ⥬ ↑ Yawns	Propofol, thiopental, sleepiness
Oshima (2007) [43]	Retrospective cohort	3a	1322 patients undergoing elective surgery, thiopental, yawning response in 461. Inhibited yawning response by thiopental + fentanyl (*n* = ~ 450), female sex, clonidine	During induction, around 50% of thiopental-induced patients (not treated with fentanyl) yawned	↑ Obstructive airway ⥬ ↑ Yawns	Propofol, thiopental, sleepiness
Tsou (2008) [44]	Prospective cohort	2a	546 ASA 1 / 2 patients undergoing elective surgery, induced with propofol only (*n* = 386), propofol with atropine (*n* = 90), and propofol with fentanyl (*n* = 50)	During induction, yawning occurred in 54% (propofol), 61% (propofol + atropine), and 0% (propofol + fentanyl)	↑ Obstructive airway ⥬ ↑ Yawns	Propofol, sleepiness
Oshima (2010) [45]	RCT	2a	180 ASA 1/2 patients undergoing elective surgery with thiopental induction combined with landiolol (*n* = 60), nicardipine (*n* = 60), and saline (*n* = 60)	During induction, yawning occurred in 7% (thiopental + landiolol), 17% (thiopental + nicardipine), and 47% (thiopental + saline)	↑ Obstructive airway ⥬ ↑ Yawns	Thiopental, landiolol, nicardipine, saline sleepiness
Gallup, Gallup (2010) [46]	Case report	5b	2 case reports of excessive yawning	One case with sleep apnea	↑ Obstructive airway ⥬ ↑ Yawns	Sleepiness
Carra (2011) [47]	Cross-sectional study	3a	604 patients (7-17 years old) seeking orthodontic treatment	Teeth clenching is accompanied by significant difficulties of yawning in this group (tabel 3.3.3), together with snoring/OSA-like symptoms	↓ Yawns ⥬ ↑ Obstructive airway	Teeth clenching, OSA, sleepiness
Tsou (2012) * [48]	Prospective cohort	2a	82 ASA 1 / 2 patients undergoing elective surgery with propofol induction and cardiovascular and cardio-respiratory interaction was measured	During induction with propofol, yawning occurred in 65%	↑ Obstructive airway ⥬ ↑ Yawns	Propofol, sleepiness
Zaharna (2013) [49]	Case report	5a	One patient presented with sleepiness and yawning, diagnosed with OSA syndrome and improved with CPAP	Increased yawning in OSA syndrome	↑ Obstructive airway ⥬ ↑ Yawns	OSA, sleepiness
Catli (2015) [ 50]	Prospective cohort	2a	129 patients with suspected OSA syndrome were monitored during sleep and their yawns were counted during the day, divided in 2 groups: AHI < 5 (*n* = 43), and AHI > 30 (*n* = 86), and were compared with parameters (1) duration of sleep phases, (2) oxygen saturations, (3) sleep efficacies, (4) yawning frequencies, and (5) Epworth scores	Increased AHI index and lower sO_2_ during sleep led to increased yawning during day. Group 1 (AHI 22, sO_2_ 94%) 4–5 times/day, Group 2 (AHI 87, sO_2_ 92%) 10–11 times/day	↑ Obstructive airway ⥬ ↑ Yawns ↓ Obstructive airway ⥬ ↓ Yawns	Sleepiness Thinking of yawning

↑Increased/more; ↓Decreased/less; ⥬Followed by/coincides with; ASA: Amarican Society of Anesthesiology; OSA: Obstructive Sleep apnea; AHI: Apnea-Hypopnea Index

^*^Achieved via library of Utrecht University

^**^Cross-reference

^***^Only clear confounding factors mentioned, therefore incomplete

*RCT*, randomized controlled trial; *OSA*, obstructive sleep apnea; *ASA*, American Society of Anesthesiology (classification of physical status)

## Results

A total of 449 studies were identified: 138 in PubMed and 311 in Embase. Duplicates were removed, after which title and abstract were read in duplicate for the remaining 319 articles. Thirty-six studies met the inclusion criteria, of which 24 were excluded (see flowchart, Appendix 2). Twelve eligible studies from the initial search served as input for ASReviews combined with 2238 Embase results, after which one additional article was identified, none via cross-referencing.

Two articles were obtained via the Utrecht University library [38, 48]. The studies of Oshima (2007, 2010) and Tsou (2008, 2012) were written by the same authors. Most studies were incidence studies and had a level of evidence 2 according to the LEGEND table. Results regarding the relationship between airway obstruction and yawning frequency can be found in Table 1.

The following temporal relationships were identified: (1) more obstructive airway is followed by or coincides with increased yawning, (2) less obstructive airway is followed by or coincides with decreased yawning, (3) decreased yawning is followed by or coincides with more obstructive airway, and (4) increased yawning is followed by or coincides with less obstructive airway. No studies were identified relating the frequency of yawning with asthma, COPD, or atelectasis. Different patterns and intensities of yawning in relation with to airway pathophysiology were not reported.

## Discussion

Yawning is considered to be a primitive behavior, as it is widely spread across the animal kingdom, and the yawn center is located in the vital section of the brain. An important physiological function may explain its evolutionary conservation. 

This scoping review supports a relationship between (upper) airway physiology and yawning, based on temporal relationships of yawning’s frequency change and the variation in airway patency. According to seven included studies, upper airway collapse during induction of anesthesia coincides with increased yawning in a majority of patients. However, not all patients yawned during induction, which may be explained by a rapid transition to deep sleep/sedation or opiate administration. Kocaman et al. [51] observed no yawning during propofol induction of 51 patients. However, opioids (remifentanil) were also administered before observation, which is known to inhibit yawning. Teeth clenching and opioids are related to decreased yawning, and both conditions coincide with OSA-like symptoms and respiratory suppression. Whether these complications are a direct consequence of decreased yawning is not yet clear. In the study of Bartlett, post-surgery patients were stimulated to yawn at least 10 times per hour and were instructed to use a spirometer, besides normal ambulation, sigh, and other stimulations. Pulmonary complications were significantly reduced in the yawning group. This may suggest that pulmonary complications result from decreased yawning, and may potentially be prevented by stimulating yawns. A spirometer may have been a major confounding factor, therefore repeating the study without spirometer could give valuable insights.

### Limitations

Yawning is one of the most rarely studied behaviors resulting in heterogenous studies with low level of evidence. Determinants and outcomes of included studies were variable, which makes identifying relationships between studies fairly complex. Exclusion criteria were arbitrary, as “increased” and “decreased” yawning has not yet been defined and was therefore often based on the subjective view of authors. Symptoms of obstructive airway conditions varied widely and may have different relationships with yawning, but did not have objective parameters. Distinct yawn patterns and intensities were not documented. These parameters  however may also influence obstructive airway conditions, instead of only frequency of yawning. Dilator forces should be within the scope of future research, as they are never or seldom described. The temporal relationships in Table 1 should not be interpreted as causal factors yet, as multiple confounding factors are present and influence both the yawning frequency and the airway condition (e.g., low-vigilance state, thinking of yawning, and medication). Therefore, these relationships cannot be extrapolated to yawning’s function. Further research is necessary to investigate the following causal relationships: (A) decreased yawning leads to more obstructive airway, (B) increased yawning leads to less obstructive airway, (C) more obstructive airway leads to increased yawning, (D) less obstructive airway leads to decreased yawning.

We combine these modest results with yawning’s characteristics (see “Introduction”) to come to a novel hypothesis on the function of yawning.

### Airway physiology hypothesis

The airway physiology hypothesis entails:

Yawning secures long-term oxygenation by creating an enlarged airway lumen by muscle repositioning, primarily in the upper airway. A single letter-to-the-editor by Hanning, [52] which was found during this literature search, mentioned this same hypothesis; however, the mention was brief and without discussion.

**Fig. 2:**
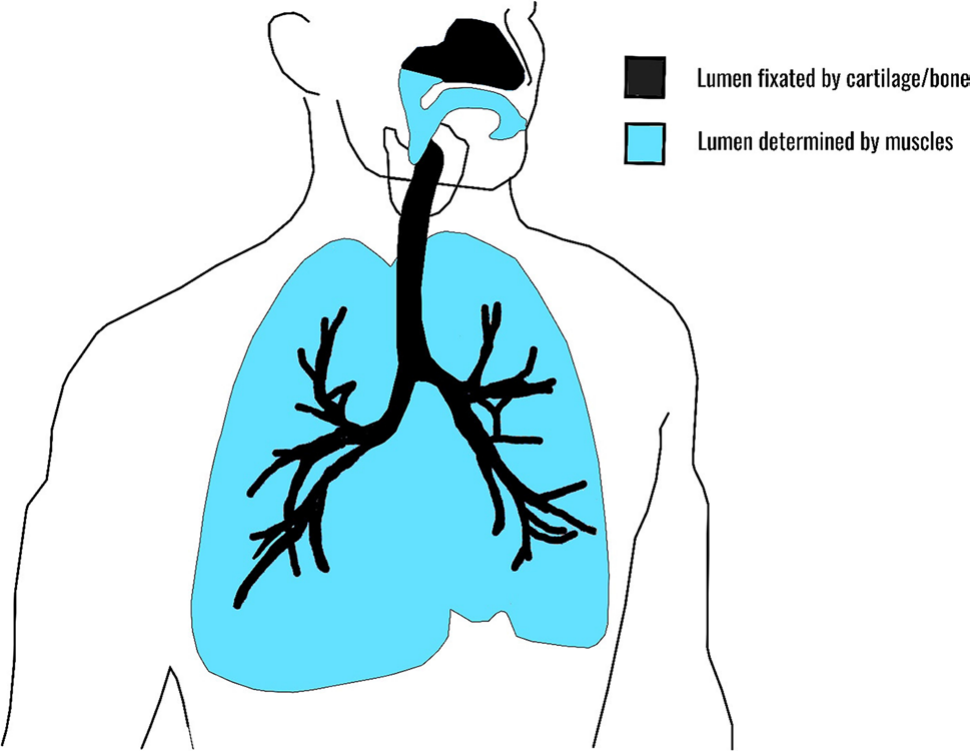
Schematic overview of the airway. The airway can be divided into two mechanical properties: (1) lumen which is relatively dilated by muscles (blue) and (2) lumen which is fixed by cartilage or bone (black). A dynamic lumen is essential for peristaltic movements, vocalization in the pharynx, and ventilation in the lungs

The airway is a hollow and dynamic passage, which must remain open at all costs. The airway lumen is continuously subject to forces of gravity, changing respiratory pressures, and body movements. Therefore, the airway is largely supported by cartilage and bony structures (black in Fig. 2). However, similar fixation of the airway would not be functional in the pharynx and lungs (blue in Fig. 2). The dynamic movements of the pharynx and lungs are essential, and distinctively mentioned below.

### Upper airway patency

**Fig. 3:**
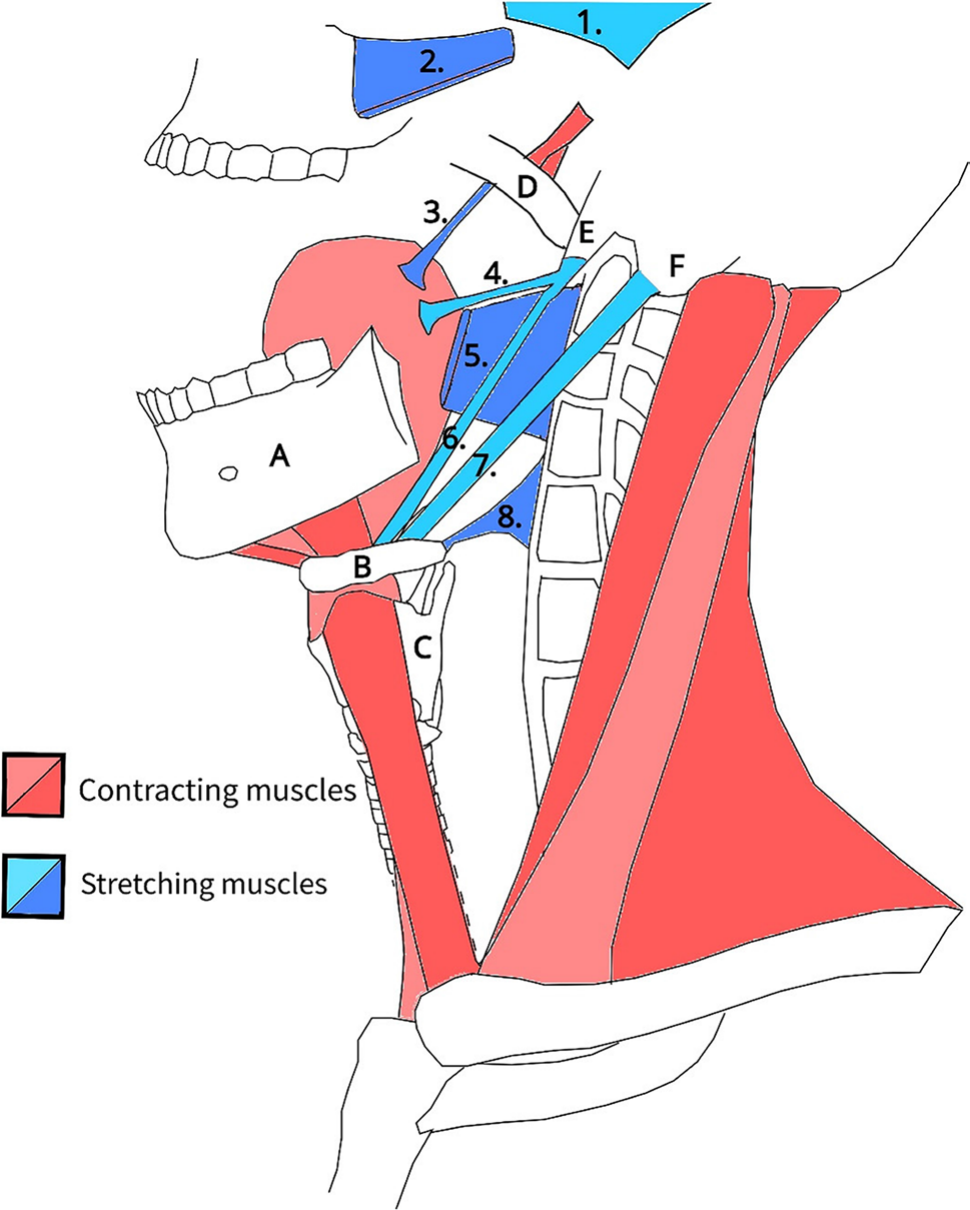
2D model of muscle movements during a yawn: contracting muscles (red) and stretching muscles (blue). During a yawn, the airway is fully dilated, most prominently in the pharynx. Maximal mouth gaping and pharyngeal widening are achieved by movement of the mandible (**A**), hyoid (**B**), larynx (**C**), and soft palate (**D**). This results in powerful stretching of jaw and pharyngeal muscles, which are essential for chewing and swallowing. The styloid process (**E**) and mastoid process (**F**) are anchor points for swallowing muscles. The stretched muscles include the temporal (1), masseter (2), palatoglossal (3), styloglossus (4), superior pharyngeal constrictor (5), stylohyoid (6), posterior belly of digastric (7), and middle pharyngeal constrictor muscle (8). The palatopharyngeal, stylopharyngeus, and inferior pharyngeal constrictor muscles are stretched as well, however not illustrated here. Stretching is achieved by contraction of antagonist muscles in three groups (Fig. 1): the floor of the mouth muscles (geniohyoid, mylohyoid, hyoglossus, and anterior bellies of the digastric), the infrahyoidal muscles (omohyoid, thyrohyoid, sternohyoid, and sternothyroid), and the pharyngeal and palatal elevators (tensor and levator veli palatine). Stretching muscles is essential to prevent tightening and loss of range of motion. According to our hypothesis, the airway is widened by a yawn and long-term oxygenation is safeguarded

Pharyngeal muscles determine the diameter and volume of the upper airway. These muscles interact in a complex fashion to constrict and dilate for different purposes: securing the airway, swallowing, and vocalization. Upper airway patency (or volume) is regulated by position of the tongue, the hyoid apparatus, the posterolateral pharyngeal walls, and the soft palate [53]. Muscles responsible for these positionings are illustrated in Fig. 3 in yawning condition. Involved muscles must be perfectly balanced, as both loose and tight pharyngeal muscles result in a disturbed balance, and could lead to higher airflow resistance, higher respiratory effort, and potential apnea [54]. Besides endangering respiration, proper swallowing and vocalization may also be at risk due to muscle imbalance. Yawning (now considered repetitive airway stretching and dilation) may be the ideal maneuver to restore this muscle balance. The entire airway dilates maximally during a yawn, which automatically results in powerful stretching of the jaw and swallowing muscles. This is illustrated in Fig. 3: in blue stretching constrictive pharyngeal muscles, and in red tightening dilator muscles. In basic muscle physiology stretching is known to help elongate muscles (prevent tightening), create comfortable and functional muscle tone, and increase range of motion [55]. This is well achieved by gradual eccentric contraction in fits of two or three with increasing intensity (see “Introduction”). Post-yawn widening is not well documented. But video-endoscopic findings of Titze [56] and Boone [22] showed that the pharyngeal lumen maintains a widened position after a yawn. This automatically decreases airway resistance and reduces the chance of collapse [53]. Significant widening and stretching during a yawn and an increase of airway lumen size post-yawning substantiate this hypothesis. However, these studies must be repeated with better imaging in the future.

In what circumstances is this muscle repositioning and pharyngeal widening favorable? Alteration of muscle tone throughout the body, including pharyngeal muscles, is a result of a changing vigilance state of the brain [53]. Drowsiness often precedes sleep, in which narrowing of the pharynx significantly increases in all individuals [53]. The upper airway is exponentially more collapsible when the airway lumen is reduced, [53] which is why adequate muscle tension in dilator muscles is required during drowsiness and sleep. Anticipatory action by dilation and muscle repositioning is therefore essential. An example of insufficient muscle tone during sleep is snoring, while more serious conditions include obstructive sleep apnea (OSA) with apneas during the night. Most extreme are situations when the brain is shut down and total collapse of the airway follows. Unconscious patients may die due to a collapsed pharynx, which is the reason why the jaw thrust, chin lift, stable side position, and intubation are life-saving maneuvers/interventions. These widely accepted principles underline the importance of dilator forces in upper airway physiology. Yawning as a key player in dilation may explain frequent yawning around drowsiness, around sleep, during induction of anesthesia, and excessive yawning in patients with OSA. On the other hand, it may also explain why decreased yawning (e.g., by teeth clenching or opioids) coincides with OSA-like symptoms, as was found in the results [57, 47].

Repositioning pharyngeal muscles is also required when particular muscles have high muscle tone after repetitive use (e.g., in chewing, swallowing, and vocalization) or top-down overstimulation (e.g., “lump in throat” due to stress, emotions) [53, 58]. Already accepted is the automatic activation of dilator muscles in the pharynx during inspiration to prevent collapse [53]. A compensating mechanism for variable muscle tone due to a variable vigilance state, repetitive swallowing, or chewing movements is not yet described. Could yawning prevent collapse by muscle repositioning in these conditions?

### Yawning trigger

What neural feedback mechanism may be responsible to anticipate on airway collapse? Pharyngeal sensory feedback via the vagal nerve was suggested by Evans (1978), who described two cases of choking children who yawned frequently, and stopped yawning after obstruction was relieved. We suggest that proprioceptive feedback from pharyngeal muscles is essential information to orchestrate a complex muscle balance. Proprioceptive feedback responsible for triggering a yawn is demonstrated by voluntarily opening the jaw repetitively and inhaling gradually (introduction). This imitation alters pharyngeal muscle tones, temporarily narrows the airway, and triggers a yawn in most individuals [3, 19, 22]. Other triggers of yawning discussed in the “Introduction” may also result from reduced muscle tone and accompanying proprioceptive feedback. If extrapolated, the beginning of a yawn might even be the trigger for continuation of a yawn (or the “real yawn”). Relative frequent yawning of children may also be explained by their relative narrow airway, which makes frequent anticipation crucial. Whether or not there is a relationship of airway patency (indirectly implicating yawning) and sudden infant death syndrome (SIDS) may be a scope of future research.

### Yawning and atelectasis

In the lungs, compliance is essential for ventilation by alternate expansion and shrinkage. This compliant property may, however, also lead to bronchiolar and alveolar collapse (atelectasis). This happens during sleep, when shallow breathing and relative hypoventilation are common due to lowered oxygen demand [59]. Deep inspiration during a yawn, in which lung volume increases 300–400% when compared to tidal volume, [26] pops-open the small airways in the lungs and secures long-term oxygenation. This was also suggested by Cahill et al. (1978) and Walusinski et al. (2006). Bartlett et al. [38] stated that yawning is functional in preventing or treating atelectasis by prolonged maximal inflation. Sighing and coughing are accepted maneuvers to prevent and treat atelectasis. However, the yawn maneuver needs further investigation to imply such a purpose. Preventing atelectasis may also be interpreted as a minor function of yawning, comparable to non-verbal communication, mid-ear pressure clearing, and other advantageous “side effects.”

### Fetal yawning

Another important feature of yawning may affect fetuses, who yawn frequently from the first trimester to birth. Provine (2005) proposed that fetal yawning may sculp the jaw joint, while others stated it may help in spreading the surfactant in utero. Some stated it may only serve as preparation for later life [19]. To extend our hypothesis, fetal yawning may help airway development by repetitive dilation and muscle repositioning. The airway must be in perfect condition to anticipate on one of the most critical moments in life: the first breath.

### Evolutionary perspective

Yawning is executed by almost all, if not all vertebrates, which implies a physical function shared by all members of this group. One similarity is that all members have one hollow structure used for both dilator and constricting purposes: oxygenation by airflow or water current versus digesting by biting, chewing, and peristaltic movements. This is why this hollow structure is dynamic and uniquely modifiable by interacting muscles. These distinct physiological functions demand opposite muscle movements with limited fixation to the surrounding. Oxygenation remains the top priority, which is why strong repositioning movements must take place to restore a vital, dilated balance. If correct, one needs little imagination to realize yawning has likely been executed for millions of years, even by dinosaurs.

### Future perspective

If indeed, yawning has an important role in airway physiology, yawning will also have beneficial effects in patients with a collapsible or obstructing airway. For example, yawning may potentially counteract respiratory complications due to opioid use (which inhibits yawning) and OSA, and it may well influence swallowing disorders. To sustain these speculations, much more about yawning needs to be studied. Suggestions for further research include (1) imaging for precise determination of muscles involved before, during, and after a yawn; (2) imaging and/or flow measurements of airway resistance and volume before and after a yawn; (3) RCTs of the effect of stimulated yawns on OSA, swallowing disorders, and postoperative pulmonary complications (e.g., atelectasis); and (4) animal trials of respiratory effects resulting from absent or decreased yawning.

## Conclusion

Based on the available literature and physiological characteristics, we suggest that yawning is the ideal maneuver to reposition all muscles around the airway, thereby preserving the lumen and securing long-term oxygenation. Airway patency is therefore safeguarded by powerful muscle stretching and dilation. This hypothesis may explain increased yawning around sleep, around eating, during particular stressful events, and in children, OSA, and other conditions with a narrowed airway. Furthermore, yawning may be involved in fetal airway development, and may have beneficial effects on respiration, swallowing, and vocalization by restoring muscle balance [22, 60]. All these features of yawning may help explain its evolutionary conservation, which would make yawning one of the most underestimated physical behaviors of modern times.

## Figures and Tables

**Fig. 4:**
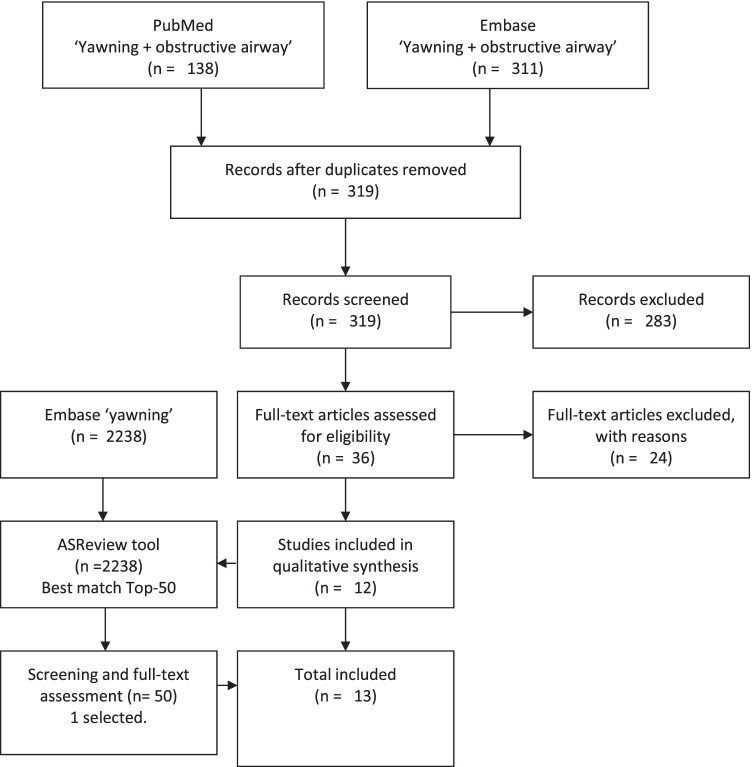
From the 449 unique publications that were found in the systematic literature search, only 12 publications were eligible for this scoping review. One additional article was found with deep learning (ASRreview tool) in a broad literature search

## Appendix 1

Table 2

**Table 2:** Database search

Medline (*n* = 138)
(Yawn*[Title/Abstract] OR “Yawning”[Mesh]) AND (airway[Title/Abstract] OR dyspnea[Title/Abstract] OR respirat*[Title/Abstract] OR breath*[Title/Abstract] OR ventilat*[Title/Abstract] OR pharyn*[Title/Abstract] OR pulmon*[Title/Abstract] OR anesthes*[Title/Abstract] OR anaesthes*[Title/Abstract] OR atelectasis[Title/Abstract] OR pneum*[Title/Abstract] OR obstruct*[Title/Abstract] OR collaps*[Title/Abstract] OR apnea[Title/Abstract] OR asthma[Title/Abstract] OR COPD[Title/Abstract] OR “Pharynx”[Mesh] OR “dyspnea”[Mesh] OR “Asthma”[Mesh] OR “Apnea”[Mesh] OR “Pulmonary Disease, Chronic Obstructive”[Mesh] OR “Pulmonary Atelectasis”[Mesh] OR “Anesthesia”[Mesh])
Embase (*n* = 311)
(airway:ab,ti,kw OR respirat*:ab,ti,kw OR ventilat*:ab,ti,kw OR breath*:ab,ti,kw OR pharyn*:ab,ti,kw OR pulmon*:ab,ti,kw OR anesthes*:ab,ti,kw OR anaesthes*:ab,ti,kw OR atelectasis:ab,ti,kw OR pneum*:ab,ti,kw OR obstruct*:ab,ti,kw OR collaps*:ab,ti,kw OR apnea:ab,ti,kw OR asthma:ab,ti,kw OR copd:ab,ti,kw OR “asthma”/exp OR “apnea”/exp OR “dyspnea”/exp OR “chronic obstructive lung disease”/exp OR “atelectasis”/exp OR “anesthesia”/exp OR “pharynx”/exp) AND (yawn*:ab,ti,kw OR “yawning”/exp)
ASReviews – Embase (*n* = 2272)
(yawn*:ab,ti,kw OR “yawning”/exp)
